# Identification of Non-Volatile Compounds That Impact Flavor Disliking of Whole Wheat Bread Made with Aged Flours

**DOI:** 10.3390/molecules27041331

**Published:** 2022-02-16

**Authors:** Wen Cong, Edisson Tello, Christopher T. Simons, Devin G. Peterson

**Affiliations:** Department of Food Science and Technology, The Ohio State University, 2015 Fyffe Rd., Columbus, OH 43210, USA; cong.28@osu.edu (W.C.); tellocamacho.1@osu.edu (E.T.); simons.103@osu.edu (C.T.S.)

**Keywords:** consumer liking, whole wheat flour, untargeted LC/MS profiling, flour storage, phosphocholine

## Abstract

Whole wheat flour has a shorter shelf life than refined wheat flour due to off-flavor development. An untargeted liquid chromatography/mass spectrometry (LC/MS) flavoromics approach was applied to identify compounds that negatively impact the flavor liking in whole wheat bread made from aged flours. The chemical profiles of thirteen breads made from aged flours were obtained using LC/MS and modeled by orthogonal partial least squares (OPLS) to predict flavor liking. Top predictive chemical features (negatively correlated) were identified as pinellic acid (9*S*,12*S*,13*S*-trihydroxy-10*E*-octadecenoic acid), 12,13-dihydroxy-9*Z*-octadecenoic acid, and 1-(9*Z*,12*Z*-octadecadienoyl)-*sn*-*glycero*-3-phosphocholine. The sensory analysis confirmed the three compounds increased the bitterness intensity of the bread samples. The formation of the trihydroxy fatty acid bitter compound, pinellic acid (9*S*,12*S*,13*S*-trihydroxy-10*E*-octadecenoic acid), was impacted by the lipoxygenase activity of the flour; however, there was no influence on the formation of 12,13-dihydroxy-9*Z*-octadecenoic acid or 1-(9*Z*,12*Z*-octadecadienoyl)-*sn*-*glycero*-3-phosphocholine. Additionally, the concentrations of all bitter compounds were significantly higher in bread made from aged flour versus non-aged flour.

## 1. Introduction

The health benefits of whole grain intake have been linked to a decreased risk of weight gain and a reduction in chronic pathological conditions including heart disease, cancer, and diabetes [1,2]. Consumers are becoming increasingly aware of the contribution of whole grains to a healthy diet; however whole grain intake on average is still far below the recommended levels [3], mainly due to the negative flavor attributes such as oxidized aroma notes and bitter taste due to lipid oxidation [4,5]. 

Flour can be stored for months prior to utilization due to distribution logistics. Generally, wheat flour has a use-by date of 3–9 months after milling [6]. Lipids are one of the most chemically unstable food components during storage, and deterioration produces oxidative compounds, which can be perceived as off-flavors [7]. Endogenous lipids, though a minor component in whole wheat flour, contribute substantially to flour functionality. The impact of lipid oxidation on the shelf life of whole wheat flour and on the flavor profile of whole wheat products has been extensively studied [6,8]. Hydrolytic and oxidative lipid degradation have been shown to affect both the taste and aroma acceptability of whole wheat bread, the associated off-flavors being characterized as musty, bitter, and rancid [9]. Additionally, lipoxygenase enzymes are well-known to catalyze lipid oxidation forming aversive volatile flavor molecules such as ketones, lactones, furans, etc. [10]. The sensory acceptability (taste and aroma) of whole wheat bread was reported to be inversely related to the concentration of free fatty acids in whole wheat flour [11]. Volatile compounds generated during flour storage, which are products of secondary lipid oxidation, are associated with undesirable sensory attributes such as rancidity [9]. Lipoxygenase is also known to impact the generation of pinellic acid, which has been identified as a key contributor to bitterness in whole wheat bread [12,13]. Additionally, Jiang and Peterson identified several bitter compounds in whole wheat bread crust, such as the Maillard reaction products pyrrole, pyranone, and short chain fatty acid derivatives [14].

There is limited information regarding the influence of non-volatile compounds on consumer flavor liking of whole wheat bread, particularly as impacted by flour storage. The overall aim of this project was to identify compounds that negatively impact consumer acceptability in thirteen bread samples made with aged whole wheat flour using an untargeted liquid chromatography/mass spectrometry (LC/MS) chemical profiling flavoromics approach. Highly predictive compounds of liking were purified using Prep-LC and further evaluated by a sensory recombination testing. Additionally, the impact of flour lipoxygenase on the generation of compounds predictive of flavor liking was investigated.

## 2. Results and Discussion

### 2.1. Consumer Acceptance Test

**Figure 1:**
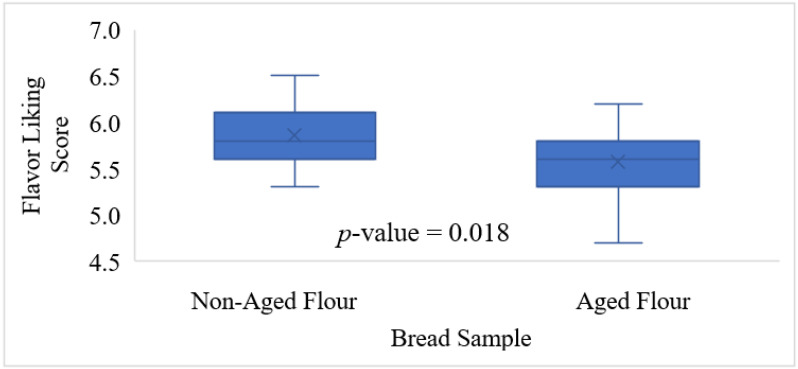
Average consumer flavor liking scores (1–9) of whole wheat bread samples (*n* = 97) made with non-aged and aged whole wheat flour. Boxplots present the median correct responses (solid line in the middle), upper and lower quartiles, mean correct responses (X marks), minimum correct responses (lower whisker), and maximum correct responses (upper whisker). The significant difference was assessed using Student’s *t*-test.

The impact of flour storage on the flavor liking scores of bread samples made with thirteen different sourced hard wheat samples is shown in Figure 1. When the bread was made with the aged flour, a subtle but significant decrease was observed, with the average score decreasing from 5.9 to 5.6 (*p* < 0.05). Therefore, the chemical changes in whole wheat flour induced during aging impacted the product liking as anticipated. 

**Figure 2:**
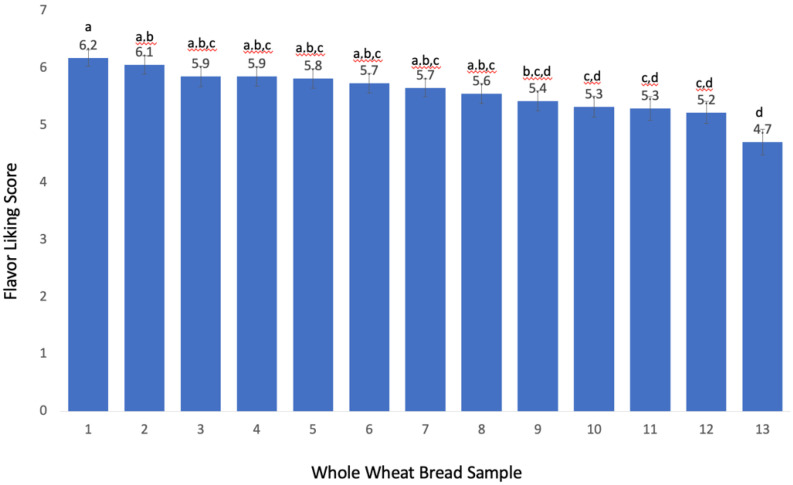
Average consumer flavor liking scores (*n* = 97) and standard errors of whole wheat bread samples made with aged flour; different letters are significantly different (*p*-value < 0.05) according to Tukey’s HSD.

The individual flavor liking scores of thirteen whole wheat bread samples are also shown in Figure 2 and ranged from 4.7 (“dislike slightly” to “neither like nor dislike”) to 6.2 (“like slightly” to “like moderately”), and significant differences of flavor liking were observed between the samples (two-way ANOVA, *p*-value < 0.05). In comparison to overall liking, flavor liking was the most correlated (r = 090), followed by texture (r = 0.80), color (r = 0.58), and the aroma (0.54). This result highlights the important role flavor has on consumers’ overall liking of whole wheat bread.

### 2.2. Untargeted Chemical Profiling Using LC/MS 

**Figure 3:**
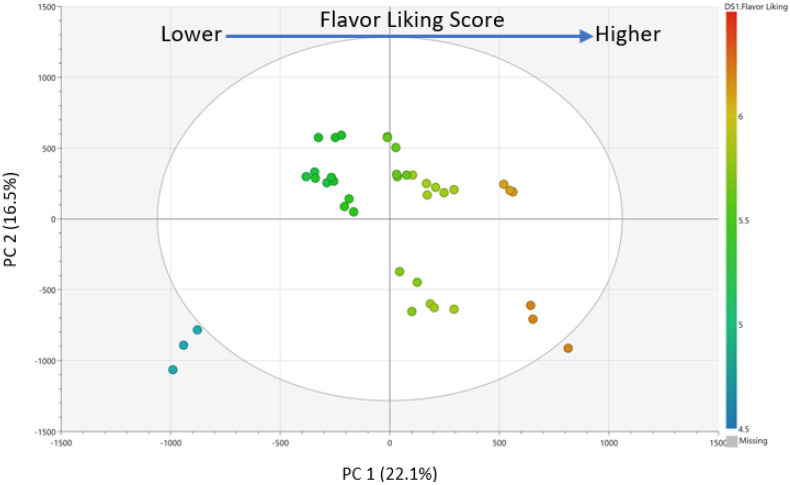
Scores scatter plot of OPLS regression model (Pareto scaling) for the consumer overall liking scores (*y*-variable, *n* = 97) and LC/MS chemical profiling data (*x*-variable, *n* = 791) from thirteen whole wheat bread samples in triplicate. Model quality was R^2^Y = 0.98 and Q^2^ = 0.96. Samples are colored by liking score values.

The chemical profiles of the thirteen bread samples were analyzed by untargeted LC/MS techniques to investigate compounds that impacted flavor liking. After data processing and peak picking, a total of 791 chemical features, defined by retention time by mass-to-charge (RT_*m/z*), were selected to build statistical models predictive of flavor liking. A review of an unsupervised principal component analysis (PCA) model indicated no outliers and good reproducibility. A supervised orthogonal partial least square (OPLS) model was subsequently utilized to select highly predictive features of flavor liking based on predictive variable of importance (VIPpred) scores. The OPLS model regression (Figure 3) had a high model quality with a R^2^Y (>0.98) and Q^2^ (>0.96) indicating an excellent fit and high predictive ability. A review of the score scatter plot showed good separation of samples, based on liking scores by predictive PC (PC1). Additionally, permutation testing indicated the model was not overfitting (Permutated R^2^ = 0.26, Q^2^ = −0.68).

Based on the OPLS model, the five most predictive chemical features (RT_*m/z*) based on VIP predictive scores [15] were selected for further evaluation (shown in Table 1). All five features were negatively correlated with liking, indicating that acceptance of whole wheat bread was influenced to a greater degree by aversive flavor attributes. People tend to weigh negative information more heavily than positive information, which has been termed negative bias [16].

### 2.3. Identification of Predictive Compounds of Flavor Disliking 

Among the five top predictive chemical features (Table 1), three were successfully isolated in high purity (>90%) from whole wheat bread using multi-dimensional LC to provide standards for quantification, sensory analysis, and structural elucidation, which included (RT_*m/z*) 4.19_329, 7.02_313, and 8.66_564. The other two chemical features (RT_*m/z*) 9.11_295 and 9.26_295 were unsuccessfully purified; however, they were tentatively identified based on their MS accurate mass and fragmentation patterns. Interestingly, only two of the top highly predictive compounds in Table 1 (VIP score > 3) were similarly reported for whole wheat bread made with non-aged flour [13]. Specifically, features (RT_*m/z*) 9.11_295, 9.26_295, and 7.02_313 were selected for whole wheat bread made with aged flour only, indicating that the chemical profile impacting flavor liking of whole wheat bread was impacted by flour aging.

**Figure 4:**
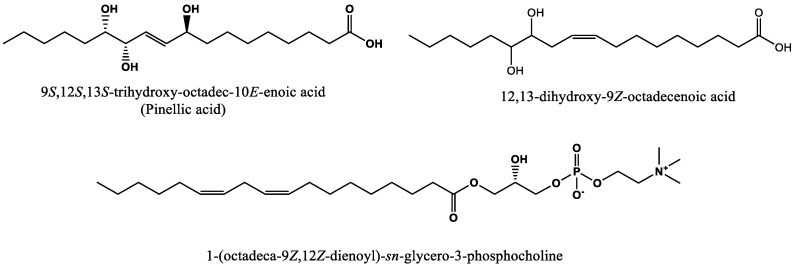
Chemical structures of three highly predictive compounds of whole wheat bread flavor disliking made with aged flour.

The most predictive chemical feature (RT_*m/z*) 4.19_329 (negatively correlated, VIP score of 13.3) was analyzed by accurate mass LC/MS-quadrupole time of flight (QToF) analysis reported an *m/z* 329.2326 [M–H]^–^ and elemental composition of C_18_H_33_O_5_ (∆0.6 ppm) and was identified as 9*S*,12*S*,13*S*-trihydroxy-10(*E*)-octadecenoic acid, pinellic acid (Figure 4) after MS/MS fragmentation matched the authentic standard (see Supplemental Material, Figure S1). Previously, pinellic acid was also reported as the most predictive compound of flavor disliking in whole wheat bread made with the non-aged flour [13]. Therefore, pinellic acid played an important role in the whole wheat bread liking regardless of flour aging. Bin and Peterson [12] have reported that pinellic acid was the main contributor to bitterness in whole wheat bread crumb. 

The second chemical feature isolated (RT_*m/z*) 7.02_313 (negatively correlated, VIP score 5.1) was analyzed by accurate mass LC/MS-QToF analysis, showed an m/z 313.2379 [M–H]^−^ with an elemental composition of C_18_H_33_O_4_ (∆1.3 ppm), and was identified as 12,13-dihydroxy-9(*Z*)-octadecenoic acid (Figure 4) after MS/MS fragmentation comparison with the literature [17] and authentic standard (Supplemental Material, Figure S2). This compound was only identified as a top predictive compound for whole wheat bread made with aged flour compared with bread made with non-aged flour [13]. It was postulated that 12,13-dihydroxy-9(*Z*)-octadecenoic acid was a derivative of linoleic acid, and it has been suggested to act as an antifungal substance in plants [18]. This compound has been identified in spring and winter wheat varieties and has been suggested as a lipid oxidation product in grains [19]. 

The third chemical feature isolated (RT_*m/z*) 8.66_564 *m*/*z*, (negatively correlated, VIP score 3.0) was analyzed by accurate mass LC/MS-QToF analysis with an accurate mass of *m/z* 564.3313 [M+FA–H]^−^ and elemental composition of C_27_H_51_O_9_NP (∆2.1 ppm) and was identified as 1-(9*Z*,12*Z*-octadecadienoyl)-*sn*-*glycero*-3-phosphocholine (LPC 18:2) (Figure 4) based on the literature and by MS/MS using an authentic standard (Supplemental Material, Figure S3). This compound was previously reported in whole wheat bread made with non-aged flour [13]. These glycerophospholipids are ubiquitous and known to play a crucial role in the cell lipid bilayer membrane [20]. Polar lipids such as LPC are known to have stabilizing effects on gas cells that can impact the bread loaf volume [21].

The remaining two chemical features (RT_*m/z*) 9.11_295 and 9.26_295 (Table 1) were tentatively identified by accurate mass LC/MS-QToF MS/MS analysis as 10(*E*),12(*Z*)-9-hydroxyoctadecadienoic acid (9-HODE) and 9(*Z*),11(*E*)-13-hydroxyoctadecadienoic acid (13-HODE). The MS/MS fragmentation (collision energy 15 V, Supplemental Material, Figure S4) included *m/z* 277 and 171 for 9-HODE and *m/z* 277 and 195 for 13-HODE and were in agreement with the fragmentation patterns reported in the literature [19]. In addition, 9-HODE and 13-HODE are widely distributed linoleic acid metabolites generated through the lipoxygenase pathway [22]. The mixture of 9-HODE and 13-HODE has been reported as bitter compounds in stored oat flour and whole wheat bread [23,24].

### 2.4. Quantification of Compounds Predictive of Flavor Disliking

**Figure 5:**
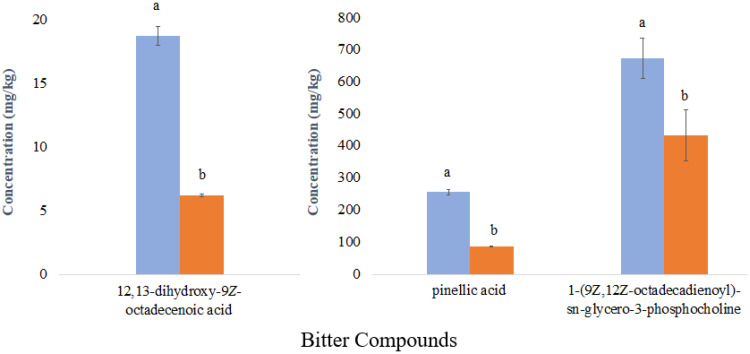
Concentrations and standard errors of three predictive compounds of wheat bread of flavor disliking; most liked sample (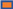) and least liked sample (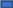); different letters indicate significant differences according to Student’s *t*-test (*p* < 0.05).

The concentration of pinellic acid, 12,13-dihydroxy-9*Z*-octadecenoic acid, and 1-(9*Z*,12*Z*-octadecadienoyl)-*sn*-*glycero*-3-phosphocholine among thirteen whole wheat breads made with aged flour ranged from 58.5 to 257.6 mg/kg, 5.31 to 26.56 mg/kg, and 376.5 to 701.1 mg/kg, respectively. A comparison of the concentrations of the three bitter compounds between the most liked bread sample (score of 6.2, Sample #1 Figure 2) and the least liked bread sample (score of 4.7, Sample #13 Figure 2) is shown in Figure 5. The concentrations of all three compounds were significantly higher in the least liked sample (*p* < 0.05) as expected from the negative correlation from the OPLS model.

### 2.5. Sensory Analysis of Predictive Compounds of Flavor Disliking 

To validate the sensory impact of the three predictive compounds on flavor disliking, a series of sensory tests was conducted, including an evaluation of the direct taste activity of each compound, the determination of the taste recognition thresholds, and finally a recombination study in which liking was assessed in bread samples to which the compounds were added. 

#### 2.5.1. Taste Activity of Predictive Compounds of Flavor Disliking

The purified compound 12,13-dihydroxy-9(*Z*)-octadecenoic acid was dissolved in water (26.56 mg/L) to the concentration quantified in whole wheat bread sample 13 (Figure 5). Panelists reported this compound as having a bitter taste, and to the best of our knowledge, this is the first report of the flavor attributes of 12,13-dihydroxy-9(*Z*)-octadecenoic acid. Both pinellic acid and 1-(9*Z*,12*Z*-octadecadienoyl)-*sn*-*glycero*-3-phosphocholine were previously reported to be bitter compounds [13].

#### 2.5.2. Recognition Threshold Values of Compounds Predictive of Flavor Disliking

The bitter recognition threshold concentration for 12,13-dihydroxy-9(*Z*)-octadecenoic acid was determined to be 0.0038 mmol/L (1.2 mg/L). The threshold values for pinellic acid and 1-(9*Z*,12*Z*-octadecadienoyl)-*sn*-*glycero*-3-phosphocholine were previously reported at 0.058 mmol/L (19.2 mg/L) and 0.046 mmol/L (23.7 mg/L), respectively [13,25]. The concentrations of all three compounds, across the thirteen whole wheat bread samples, were above the bitter threshold values, indicating a contribution to bitter perception and the negative effect on the flavor liking. 

#### 2.5.3. Bitterness Analysis of Recombination and Bread Samples

To further validate the effects of the three compounds on the bitter perception of whole wheat bread, a recombination analysis was conducted comparing the bitterness intensity (2-AFC) of a control bread sample directly with a treatment sample (control sample spiked with the three compounds at the levels reported in the least liked sample (see Figure 5). The treatment sample was significantly more bitter (8 out of 8, *p*-value < 0.01) than the control bread and validated the impact of the three compounds on the perceived bitterness intensity of whole wheat bread. 

Additionally, the sensory panel further evaluated the perceived bitterness (2-AFC) of the least liked and most liked whole wheat bread samples (Figure 2, samples 1 and 13). The least liked whole wheat bread sample was reported to be highly significantly more bitter (30 out of 32, *p*-value < 0.001) than the most liked bread. 

Therefore, three compounds identified as highly predictive of whole wheat bread disliking were reported at a higher concentration in lower liked bread, which was demonstrated to result in a higher perceived bitterness intensity that consequently reduced consumer liking. Bitterness is a well-known flavor attribute that is aversive and negatively impacts consumer liking [26]. 

### 2.6. Effects of Lipid Oxidation Enzymatic Activity on Bitter Compound Formation

Two of the three compounds reported to contribute to bitterness perception (Figure 4) of whole wheat bread were hydroxyoctadecanoic acids, which are known to be generated by lipoxygenase [27,28]. The impact of the flour lipoxygenase activity (Lpx) on the concentration of pinellic acid, 12,13-dihydroxy-9*Z*-octadecenoic acid, and 1-(9*Z*,12*Z*-octadecadienoyl)-*sn*-*glycero*-3-phosphocholine in wheat bread was evaluated following the method described in Section 3.7. Breads made with aged lipoxygenase knockout (Lpx KO) whole wheat flour and the wild sibling control were analyzed. The Lpx activity value in the KO flour was previously reported to be 94% lower than the wild sibling control flour sample [13]. The concentration of pinellic acid was significantly lower (*p*-value < 0.01) in bread made with aged Lpx KO flour versus the wild sibling control flour at 2.9 and 112.2 mg/kg, respectively. As expected, the flour Lpx activity impacted the formation of pinellic acid. However, the concentration of 12,13-dihydroxy-9*Z*-octadecenoic acid reported no significant difference between bread made with Lpx KO or the sibling control flour, suggesting the formation of this compound was not associated with lipoxygenase enzymes. As expected, the concentration of 1-(9*Z*,12*Z*-octadecadienoyl)-*sn*-*glycero*-3-phosphocholine was not impacted by Lpx activity. This compound is an endogenous compound in whole wheat flour and a lysophosphatidylcholine (LPC), which is one of the predominant phospholipids in endosperm [25,29]. 

### 2.7. Effect of Flour Storage on Concentrations of Compounds Contributing to Flavor Disliking

Based on the observed decrease in flavor liking of whole wheat bread made with aged versus non-aged flour (Figure 1), the average concentration of pinellic acid, 12,13-dihydroxy-9*Z*-octadecenoic acid, and 1-(9*Z*,12*Z*-octadecadienoyl)-*sn*-*glycero*-3-phosphocholine was determined for the thirteen samples for both non-aged and aged flour samples. All compounds significantly increased in bread made with aged flour (*t*-test, *p* < 0.05). Notably, 12,13-dihydroxy-9(*Z*)-octadecenoic acid increased 355% after aging (from 2.53 to 11.52 mg/kg), followed by pinellic acid (48%, increased from 61.65 to 91.63 mg/kg), and 1-(9*Z*,12*Z*-octadecadienoyl)-*sn*-*glycero*-3-phosphocholine (17%, from 471.90 to 553.30 mg/kg). It is interesting to note flour aging induced the largest increase in the concentration of 12,13-dihydroxy-9(*Z*)-octadecenoic acid and was not impacted by the flour Lpx activity. This compound could potentially serve as a marker of flour quality related to aging. 

## 3. Materials and Methods

### 3.1. Chemicals and Materials

Acetonitrile (analytical grade), isopropanol (analytical grade), methanol (analytical grade), acetone (analytical grade), and formic acid (Optima, LC/MS) were purchased from Fisher Scientific (Fair Lawn, NJ, USA). Water was purified through a Barnstead Nanopure Diamond water purification system (Thermo Scientific, Dubuque, IA, USA). 12,13-Dihydroxy-9-octadecenoic acid (purity ≥ 98%) was purchased from BOC Sciences (Shirley, NY, USA). Pinellic acid (purity ≥ 97%) was purchased from Molpot (Beacon, NY, USA). 1-(9*Z*,12*Z*-octadecadienoyl)-*sn*-glycero-3-phosphocholine was purchased from Echelon Biosciences (Salt Lake City, UT, USA). Prostaglandin F2α (purity ≥98%) was purchased from Cayman Chemical (Ann Arbor, MI, USA). Yeast, sugar, and salt were purchased from a local grocery store (The Kroger Co., Columbus, OH, USA). Thirteen samples of milled US whole hard wheat flour were gifted from Ardent Mills (Denver, CO, USA), consisting of six samples of single variety (Barlow, Vida, Linkert, Glenn, SY Soren, and Advanced) Hard Red Spring (HRS), four samples of mixed variety HRS samples, and three samples of a single variety (Snowmass, Joe, and Snowcrest) of Hard White Winter (HWW). Additionally, an HRS lipoxygenase knockout (Lpx KO) and wild-type HRS control wheat lines were sourced from Arcadia Biosciences (Davis, CA, USA) and milled by Ardent Mills.

### 3.2. Samples

#### 3.2.1. Whole Wheat Flours

Samples were milled, filled in paper bags, and underwent accelerated aging at 37 °C for 8 weeks in an incubator (Thermo Fisher Scientific), then transferred to −40 °C storage in sealed plastic containers until analysis (termed aged flour samples). A replicate set of samples was immediately stored at −40 °C (non-aged flour samples) until analysis.

#### 3.2.2. Whole Wheat Bread Sample Preparation 

As previously reported by Cong et al. [13], a modified AACC straight-dough bread-making method was utilized to make the thirteen whole wheat bread samples one day before the consumer acceptance test [30]. Ingredients included 200 g flour, 10.6 g yeast (active dry), 12.0 g sucrose, 3.0 g salt (NaCl), 6.0 g regular shortening (Crisco), and 130.0 g of water per loaf. A dough mixer (KitchenAid, Benton Harbor, MI, USA) was then used to mix ingredients for approximately 2.5 min, followed by dough fermentation for 52 min at 30 °C and 85% relative humidity in a 14.6 × 8.3 × 5.7 cm loaf pan, proofing for 25 min and 33 min, punching down the dough between proofs, and baking at 215 °C (Doyon convection oven, Menominee, MI, USA) with a beaker filled with 1 L of water for 17 min. Seven replicate loaves of bread of each wheat sample were wrapped in parchment paper and stored for 1 day prior to sensory analysis. The samples for analytical analysis were cryoground with liquid nitrogen in a spice grinder (Epica, New York, NY, USA) on the same day of sensory test and stored at −80 °C until further analysis. Three biological replicates were prepared for each bread sample. 

### 3.3. LC/MS Chemical Profiling of Whole Wheat Bread Samples

Chemical profiling methodology was previously reported by Cong et al. (2021) to extract as many chemical features as possible from the whole wheat bread sample [13]. For each sample, 4 mL 50/50 isopropanol/water and 0.1% formic acid (FA) was used to extract 1.0 g of finely ground bread powder. The mixture was shaken for 5 min at 1000 rpm using Geno Grinder (Metuchen, NJ, USA). Samples were then centrifuged at 12,879× *g* for 15 min at 4 °C. An amount of 200 μL aliquots of the supernatants was diluted with 800 μL water with 0.1% FA. Then, 1 mL of sample was loaded on an Oasis hydrophilic-lipophilic balance (HLB). Prime 96-well plate cartridge (30 mg) was then used for sample cleanup, followed by 500 μL of 5% methanol/water with 0.1% FA to wash the highly polar compounds off the cartridge and 500 μL of 95% methanol/water with 0.1% FA to elute compounds retained on the cartridge. A quality control (QC) sample was a mixture of all thirteen whole wheat bread samples. The QC sample was used as a reference for the untargeted chemical profiling, and each sample was extracted and analyzed in triplicate. 

LC/MS SYNAPT G2-S HDMS Q-ToF (Waters Co., Milford, MA, USA) and a reverse-phase Cortecs C18^+^ column (2.7 µm, 2.1 × 100 mm, Waters Co., Milford, MA, USA) were utilized for untargeted chemical profiling. Sample size was 10 μL. Column temperature was maintained at 40 °C. The mobile phase was composed of water (A), acetonitrile (B), and water with 5% FA (C) at a flow rate of 0.5 mL/min. Mobile phase gradient was as follows: 0–0.5 min, 93% A, 5% B and 2% C; 0.5–1.5 min, A 93% to 68%, B 5% to 30% and C 2%; 1.5–7.5 min, A 68% to 43%, B 30% to 55% and C 2%; 7.5–11.5 min, A 43% to 3%, B 55% to 95% and C 2%; 11.5–13.5 min hold, and back to initial conditions. The settings for the mass spectrometer were as follows: electrospray ionization (ESI) was run in negative mode with a source temperature of 120 °C and desolvation temperature of 450 °C; the capillary voltage was set to 3 KV, cone voltage 30 V, ToF scan range was 50–1200 *m*/*z*, and scan time was 0.3 s; and drying gas was 1200L/h. Leucine-enkephalin (556.2771 *m/z*) was used as a reference mass to check mass accuracy throughout the analysis. 

All samples were analyzed in random order. A blank sample (extraction solvent), column standard (a mixture of 8 paraben standards), and QC sample were injected and analyzed at the beginning of the sample sequence and after running every 10 samples to check instrumental performance.

### 3.4. Multivariate Statistical Analysis (MVA)

Progenesis QI software (Nonlinear, Durham, NC, USA) was utilized for raw chromatographic data processing (LC/MS-QToF), peak picking, and alignment. Chemical “features” were reported as retention time–mass/charge ratio (RT_*m/z*) by ion intensity. Chemical features exported from Progenesis QI were further processed by R version 3.5.2 (R Foundation, Vienna, Austria) based on the coefficient of variance (CV) of each variable and their abundance. The cutoff for CV was 20% and for abundance 500 counts.

Pareto scaling and mean-centering were performed prior to model generation. PCA (Supplemental Material, Figure S5) and OPLS (Figure 3) regression models were calculated to select chemical features driving consumer liking using SIMCA 14.0 (Sartorius Stedim Biotech, Umeå, Sweden). In the OPLS regression model, consumer overall liking scores of whole wheat bread samples were assigned as Y variable, while chemical features (RT_*m*/*z* by ion abundance) were assigned as X variables. The predictive variable of importance (VIPpred) scores and S-plot were subsequently generated to select highly significant predictive chemical features. The five most predictive chemical features were selected based on VIP predictive scores for further evaluation.

### 3.5. Off-Line Multidimensional Preparative-LC/MS Fractionation

Three untargeted LC/MS chemical features (RT_*m/z:* 4.19_329, 7.02_313, and 8.66_564) were isolated from whole wheat bread for further analysis. Finely ground bread powder (560 g) was extracted with 2240 mL 50/50 isopropanol/water and 0.1% formic acid (FA) solution in polystyrene falcon tubes using Geno Grinder (Metuchen, NJ, USA) for 5 min at 1000 rpm, followed by centrifuge at 10,528× *g* for 15 min at 4 °C. The supernatant was collected and diluted with water with 0.1% FA to 10% isopropanol solvent. Samples were cleaned up using solid phase extraction (SPE) 10 g C18 (Waters Co., Milford, MA, USA) cartridge: conditioning with 35 mL methanol, re-equilibration with 35 mL 5% methanol, loading 480 mL sample, followed by washing with 60 mL 5% methanol and eluting with 30 mL 95% methanol. The elutes were freed of solvent at 35 °C (Rocket Synergy Purge, Genevac, U.K.), lyophilized, and subsequently reconstituted in 160 mL of 50/50 isopropanol/water and 0.1% FA solution, then filtered through 0.45 mm nylon syringe filter (Millex; Millipore, Billerica, CA, USA).

First dimension separation was performed on a preparative LC/MS-tandem quadrupole (TQD) (Waters Co., Milford, MA, USA) coupled with fraction collector Waters 2767 (Waters Co., Milford, MA, USA). An Xbridge prep C18 (50 × 50 mm, 5 µm, Waters Co., Milford, MA, USA) column was used for separation. A flow rate of 100 mL/min was used with a binary gradient mobile phase consisting of 0.1% formic acid in water (A) and methanol with 0.1% FA (B). The gradient was as follows: 0–2 min, 5% B; 2–4 min 5–40% B; 4–5 min, 40–50% B; 5–15 min 50–62% B; 15–33 min, 62–80% B; 33–35 min, 80–95% B; 35–38 min, 95% B; 38–40 min, 5% B. The source temperature was 150 °C, desolvation temperature was 350 °C, capillary voltage was 3 kV, cone sample was 30 V, cone gas flow was 60 L/h, drying gas was 1200 L/h, and desolvation gas flow was 650 L/h. Solvent of each fraction was removed by evaporation (Rocket Synergy Purge, Genevac, UK) and lyophilization. Samples were then reconstituted with methanol to 500 mg/L. The purity level of each sample was analyzed on SYNAPT G2-S HDMS Q-ToF (Waters Co., Milford, MA, USA) in MS scan mode in positive and negative ESI.

Second-dimension fractionation was performed on Xbridge prep Shield RP18 (10 × 250 mm, 5 µm) column (Waters Co., Milford, MA, USA) to further purify samples to greater than 90% purity. The mobile phase was maintained at a flow rate of 7 mL/min using a binary solvent system of 0.1% formic acid in water (A) and in methanol with 0.1% formic acid (B). The elution gradient optimized for chemical feature (RT_*m/z*) 4.19_329 started at 58% B (0–13 min), 58–95% B (13–15 min), held for 3 min (15–18 min), and re-equilibrated at 58% B (18–20 min). For chemical feature (RT_*m/z*) 8.66_564: the elution gradient started at 60% B (0–15 min), 60–95% B (15–16 min), held for 2 min (16–18 min), and re-equilibrated at 60% B (18–20 min). For chemical feature (RT_*m/z*) 7.02_313 (RT_*m/z*): the elution gradient started at 55% B (0–15 min), 55–95% B (15–16 min), held for 2 min (16–18 min), and re-equilibrated at 55% B (18–20 min). Fractions were removed of solvent by evaporation (Rocket Synergy Purge, Genevac, UK) and lyophilization prior to further analysis.

### 3.6. Compound Identification

Analytes were identified by accurate mass and fragmentation pattern analysis using an Acquity UPLC coupled to a SYNAPT G2-S LC/MS-QToF (Waters Co., Milford, MA, USA). A Cortecs UPLC C18^+^ 1.6 µm column (50 × 50 mm, Waters Co., Milford, MA, USA) was kept at 40 °C. A flow rate of 0.5 mL/min with a tertiary gradient mobile phase consisting of solvent (A) nanopure water, (B) acetonitrile, and (C) 5% formic acid in water was used. The gradient was as follows: 0–0.75 min, B 5%; 0.75–8 min, B 5–95%; 8–9 min, B 95%; 9–10 min, B 5%; with 0–10 min, C 2%. Electrospray ionization was run in negative mode, source temperature at 130 °C with desolvation gas temperature at 400 °C, and desolvation gas flow at 800 L/h. Cone gas flow was at 150 L/h. The capillary voltage was 3 kV, and the sample cone voltage was 30 V. The collision energy was 15 V. Positive identification was confirmed after comparison with commercial standard.

### 3.7. Quantification Predictive Compounds by LC/MS-Tandem (LC/MS/MS)

One gram of bread samples in triplicate (Section 3.3) was extracted using a mixture of 1:1 isopropanol/water with 0.1% formic acid (900 µL, *v*/*v*) in 2 mL Eppendorf tubes. Then 100 µL prostaglandin F2α (10 µg/mL final concentration) was also added as internal standard. Geno Grinder was used to homogenize the sample at 1000 rpm for 10 min, followed by centrifuge at 10,528× *g* for 5 min. For compound pinellic acid and 12,13-dihydroxy-9*Z*-octadecenoic acid, 100 µL of supernatant was diluted with 500 µL water with 0.1% formic acid and further passed through a 96-well Oasis HLB plate (1 cc, 30 mg sorbent): 500 µL loading, 500 µL of 95% methanol elution. Sample clean-up of compound pinellic acid, 1-(9*Z*,12*Z*-octadecadienoyl)-*sn*-*glycero*-3-phosphocholine, and 12,13-dihydroxy-9*Z*-octadecenoic acid was slightly modified to improve the recovery. Therefore, 10 µL) of the supernatant was diluted 1:500 with water with 0.1% formic acid. A 96-well BEH C18 plate (1 cc, 40 mg sorbent) was utilized for sample clean up via a pass-through method (500 mL load and 500 mL of methanol for elution).

Quantification was carried out using 5-point standard addition calibration curves (in triplicate) to account for compound recovery and displayed good linearity for all the compounds: R^2^ > 0.99. An internal standard was used to adjust for instrument variation. The compounds quantified included pinellic acid, 1-(9*Z*,12*Z*-octadecadienoyl)-*sn*-*glycero*-3-phosphocholine, and 12,13-dihydroxy-9*Z*-octadecenoic acid. Quantitative analysis was conducted using an Acquity H-class UPLC system (Waters Co., Milford, MA, USA) coupled with a Xevo TQ-S mass spectrometer (Waters Co., Milford, MA, USA) in multiple reaction monitoring (MRM) acquisition mode. Compounds were separated using reverse-phase BEH C18 (2.1 × 50 mm, 1.7 μm, Waters Co., Milford, MA, USA) at 40 °C. A flow rate of 0.5 mL/min was used with a binary gradient mobile phase consisting of solvent (A) nanopure water with 0.1% formic acid and (B) acetonitrile with 0.1% formic acid. The gradient was as follows: 0−0.5 min, 5% B; 0.5−1.5 min, 5−35% B; 1.5−7.5 min, 35–55% B; 7.5−9 min, 55−70% B; 9−10 min, 70–95% B; 10–11 min, 95% B; and then equilibrated in the initial condition for 1 min. MS data were collected using multiple reaction monitoring (MRM) mode using the following conditions: capillary voltage of 2.2 kV, sample cone voltage of 50 V, ESI^−^, drying gas was 1200 L/h, a source temperature of 150 °C, and a desolvation temperature of 550 °C. The internal standard MS/MS transition used for prostaglandin F2α (10 µg/mL final concentration, internal standard) was ESI^−^ *m*/*z* 353→309 (collision energy 22 eV). MS/MS transitions of 12,13-dihydroxy-9Z-octadecenoic acid, pinellic acid, and 1-(9*Z*,12*Z*-octadecadienoyl)-*sn*-*glycero*-3-phosphocholine were: ESI^−^ *m*/*z* 313→183 (collision energy 22 eV), 329→211 (collision energy 22 eV) and 504→279 (collision energy 20 eV), respectively.

### 3.8. Sensory Analysis

#### 3.8.1. Consumer Acceptance Test of Whole Wheat Bread

Ninety-seven participants (73 female, 24 male), recruited by the Sensory Evaluation Center at The Ohio State University, participated in a consumer liking study of 13 whole wheat breads through an online screening questionnaire. Participants were recruited who primarily consumed whole wheat bread (average consumption was one or more times per week) and were willing to attend two testing sessions spaced approximately 1-week apart.

Consumer acceptance testing was performed following the same method as described in previous work by Cong et al. [13] and was conducted on two days over one week using a complete block design. A total of 13 whole wheat breads were evaluated by each participant (session 1 *n* = 6, session 2 *n* = 7). Whole wheat breads were made one day before the consumer acceptance test and stored at room temperature wrapped in parchment paper before testing. The sample size was 3 cm × 2 cm × 1 cm (L × W × H) squares that contained both crust and crumb of the bread. Samples were cut approximately 1.5 h before testing. Two squares of bread were served in 2 oz (59 mL) clear-lidded soufflé cups labeled with a 3-digit code. Within each session, the serving order of each bread sample was balanced. Water was used as a palate cleanser. There was a 1 min break between samples. Participants rated the samples using a 9-point hedonic scale ranging from 1 (dislike extremely) to 9 (like extremely). Participants evaluated overall liking, as well as liking of the flavor, aroma, color, and texture. Before tasting, participants were asked to smell the sample and evaluate the liking of the aroma. When tasting the sample, participants were instructed to bite from the crust side of the sample, ensuring both crust and crumb were tasted together.

Compusense Cloud Software version 5.2 (Compusense, Guelph, ON, Canada) was used for data collection. Approval of the sensory evaluation protocol was granted by the Ethics Committee, The Ohio State University (IRB #2017E0804).

#### 3.8.2. Flavor Activity of Negatively Correlated Compounds of Flavor Liking

A consensus panel of six experienced sensory panelists was used to assess the flavor attributes of pinellic acid, 12,13-dihydroxy-9Z-octadecenoic acid, and 1-(9*Z*,12*Z*-octadecadienoyl)-*sn*-glycero-3-phosphocholine. The purified compounds were dissolved in water at the average concentration quantified in the least liked bread samples. One-milliliter samples were evaluated by panelists with nose clips.

#### 3.8.3. Human Taste Recognition Thresholds Test

The bitter recognition threshold concentrations were determined by performing a series of two-alternative forced-choice (2-AFC) tests [31]. Ten panelists were trained weekly to become familiar with the sensory methodologies used and to be able to evaluate aqueous reference solutions of bitter compounds. The panelists gave informed consent to participate and had no history of known taste disorders. Sensory analyses were performed in a sensory panel room using nose clips to prevent cross-modal interactions with olfactory cues. Prior to sensory analysis, the isolated compounds were confirmed to be effectively free of solvent traces by lyophilization. For each 2-AFC test, 2 mL of each sample was presented in pairs to the panelists. One sample was a control (water) and the other a test sample (compound dissolved in water). The samples were presented in ascending concentrations. The concentrations tested for 12,13-dihydroxy-9Z-octadecenoic acid, pinellic acid, and 1-(9*Z*,12*Z*-octadecadienoyl)-*sn*-*glycero*-3-phosphocholine were from 0.6 to 37.5 mg/kg, 8.1 to 515.2 mg/kg, and 10.5 to 674.0 mg/kg, respectively. For each 2-AFC test, panelists were asked to identify the sample that was perceived as more bitter. Pairs continued to be evaluated until the panelist identified the test sample as most bitter in two consecutive pairs of the same concentration. Panelists were asked to rinse their mouth with water between samples.

The geometric means of the last missed and first correctly identified concentrations were calculated as the best-estimate thresholds (BET) of each panelist. The taste threshold of the sensory group was the geometric mean of BET of the individual assessors. Panelists were asked to evaluate samples for two independent sessions. The values between the two sessions differed by no more than plus or minus one dilution step. Approval of the sensory evaluation protocol was granted by the Ethics Committee, The Ohio State University (IRB # 2020B0073).

#### 3.8.4. Bitterness Analysis of Recombination Samples and Bread Samples

Changes in sample bitterness intensity were evaluated by a 2-AFC test conducted to validate the causality relevance of compounds negatively correlated with flavor liking of whole wheat bread. Eight participants were recruited by the Flavor Research and Education Center (FREC) at The Ohio State University. Panelists only participated in one session, evaluating one pair of whole wheat bread samples, and the panelists were asked to pick the most bitter sample.

In the recombination test, a control sample consisting of the most liked bread sample (score = 6.2, Figure 2) was compared with a treatment sample that consisted of the control bread spiked with three predictive compounds (pinellic acid, 12,13-dihydroxy-9Z-octadecenoic acid, and 1-(9*Z*,12*Z*-octadecadienoyl)-*sn*-*glycero*-3-phosphocholine) adjusted to the concentration levels of the least liked bread sample (score = 4.7, Figure 2), Figure 5. One 4 g piece of the sample was put into a 2 oz (59 mL) lidded cup and 500 µL of water (or water with all 3 predictive compounds) was added onto the bread samples 15 min before sensory testing using a 1 mL pipet. The serving order of each bread sample was balanced, and water was used as a palate cleanser.

Furthermore, a 2-AFC test was also utilized to determine whether there was a difference in bitterness between the most liked and least liked whole wheat breads made with aged flour. Sixteen participants were recruited by FREC at The Ohio State University. The breads were baked according to the AACC straight-dough bread-making method (Section 3.2.2). Bread samples were cut into 3 cm × 2 cm × 1 cm (L × W × H) squares that contained both crumb and crust, and stored in sealed 2 oz (59 mL) cups at room temperature until evaluation. Panelists were instructed to place the entire piece of bread in their mouth, chew for 10 s, evaluate the maximum bitter intensity perceived, and then to choose the most bitter sample. The evaluation occurred in duplicate over two sessions. Within each session, the serving order of each bread sample was balanced, and water was used as a palate cleanser.

Data collection was conducted via Compusense Cloud Software version 7.2 (Compusense, Guelph, ON, Canada). Approval of the sensory evaluation protocol was granted by the Ethics Committee, The Ohio State University (IRB # 2017H0072).

### 3.9. Statistical Analysis

SPSS Statistics Version 25 (International Business Machines Corp., Armonk, NY, USA) was used for two-way analysis of variance (ANOVA), and JMP Version 14 (SAS, Cary, NC, USA) was used for paired Student t-tests. The Smith Model [32] was used to determine that replicate evaluations from the panelists could be pooled, and a binomial analysis (1-tail) was subsequently used to test for significance in 2-AFC sensory validation studies.

## 4. Conclusions

Untargeted LC/MS profiling flavoromics analysis was demonstrated to successfully model and identify compounds that impact the flavor liking of whole wheat bread. The acceptance of bread made with aged flour was impacted by lipid-derived bitter compounds, such as pinellic acid, 12,13-dihydroxy-9(*Z*)-octadecenoic acid, and 1-(9*Z*,12*Z*-octadecadienoyl)-*sn*-*glycero*-3-phosphocholine. When flour was stored for four months (compared with non-aged flour), higher amounts of all three bitter compounds were reported in the bread samples, with the largest change (355%) for 12,13-dihydroxy-9(*Z*)-octadecenoic acid. Furthermore, the amount of 12,13-dihydroxy-9(*Z*)-octadecenoic acid in the samples was not impacted by lipoxygenase activity, indicating the complexity of off-flavor generation in wheat bread. Further understanding of the mechanisms of generation for these aversive compounds is needed to provide viable strategies for flavor improvement. 

## Figures and Tables

**Table 1:** OPLS model VIP predictive compounds of whole wheat bread disliking.

Compound	Retention Time by Mass-to-Charge Ratio (RT_*m/z*)	Vip_pred_ Score
1	4.19_329	13.3
2	9.11_295	6.1
3	9.26_295	5.2
4	7.02_313	5.1
5	8.66_564	3.0

## Data Availability

Not applicable.

## Supplementary Materials

The following supporting information can be downloaded, Figure S1: LC/MS-QToF (ESI^−^) fragmentation pattern of 12,13-dihydroxy-9(Z)-octadecenoic acid from (a) bread and (b) pure standard, Figure S2: LC/MS-QToF (ESI^−^) fragmentation pattern of pinellic acid from (a) bread and (b) pure standard, Figure S3: LC/MS-QToF (ESI^−^) fragmentation pattern of 1-(9*Z*,12*Z*-octadecadienoyl)-*sn*-glycero-3-phosphocholine from (a) bread and (b) pure standard, Figure S4: LC/MS-QToF (ESI^−^) fragmentation pattern of tentatively identified (a) 10(*E*),12(*Z*)-9-hydroxyoctadecadienoic acid and (b) 9(*Z*),11(*E*)-13-hydroxyoctadecadienoic acid, Figure S5: Scores scatter plot of PCA model (Pareto scaling) for LC/MS chemical profiling data from thirteen whole wheat bread samples in triplicate.

## Sample Availability

Samples are not available from the authors.
