# Supplementary material for: Identification of Non-Volatile Compounds That Impact Flavor Disliking of Whole Wheat Bread Made with Aged Flours
Source: Molecules. 2022 Feb 16;27(4):1331. doi: 10.3390/molecules27041331 (PMC8876722; doi:10.3390/molecules27041331)
Supplement: Supplementary file 1 [file molecules-27-01331-s001.zip › molecules-1582444-supplementary.pdf]

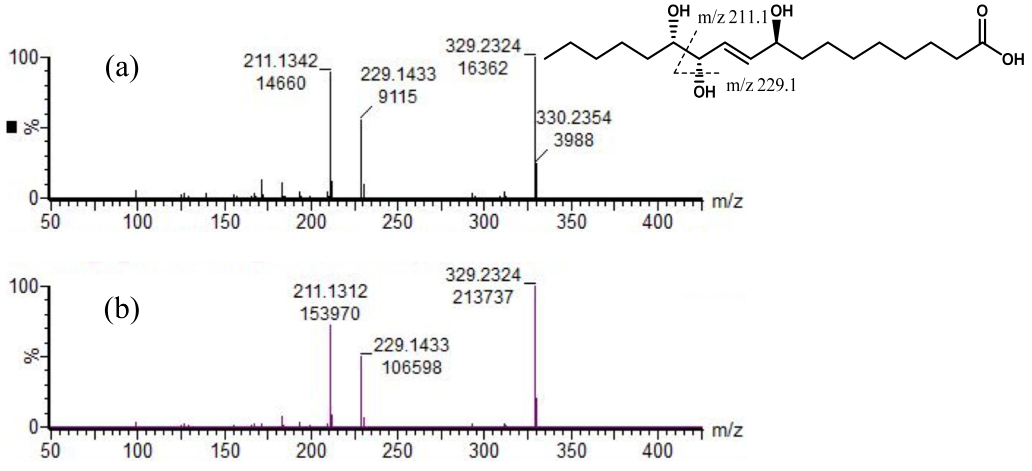

Figure S1. LC/QTOF-MS (ESI^{-}) fragmentation pattern of pinellic acid from (**a**) bread and (**b**) pure standard.

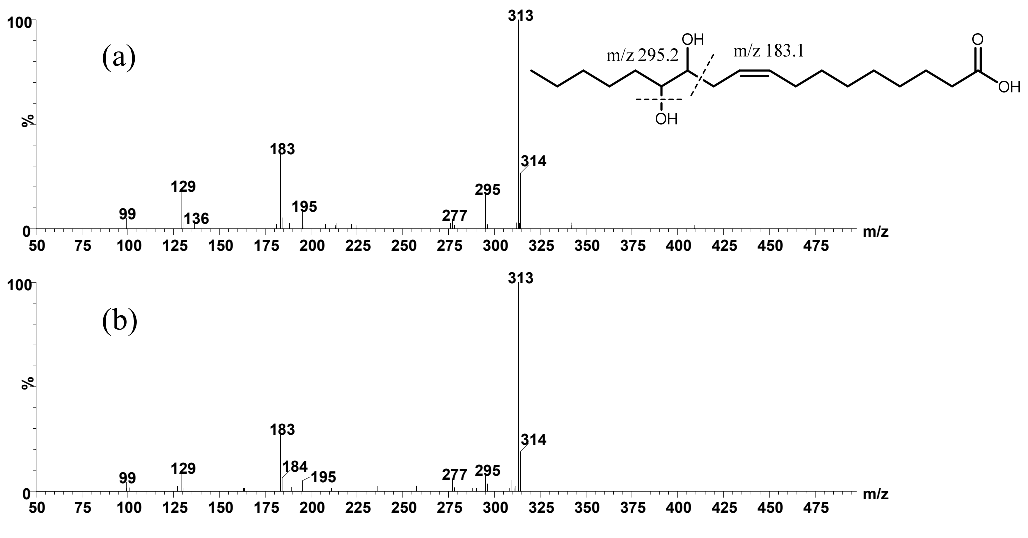

Figure S2. LC/QTOF-MS (ESI^{-}) fragmentation pattern of 12,13-dihydroxy-9(Z)-octadecenoic acid from (**a**) bread and (**b**) pure standard.

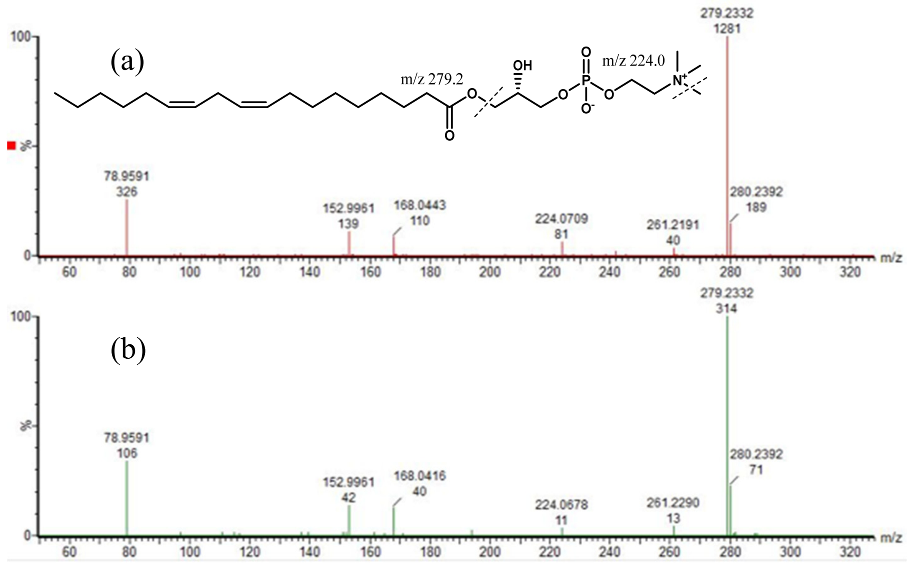

Figure S3. LC/QTOF-MS (ESI⁻) fragmentation pattern of 1-(9*Z*,12*Z*-octadecadienoyl)-*sn*-glycero-3-phosphocholine from (**a**) bread and (**b**) pure standard.

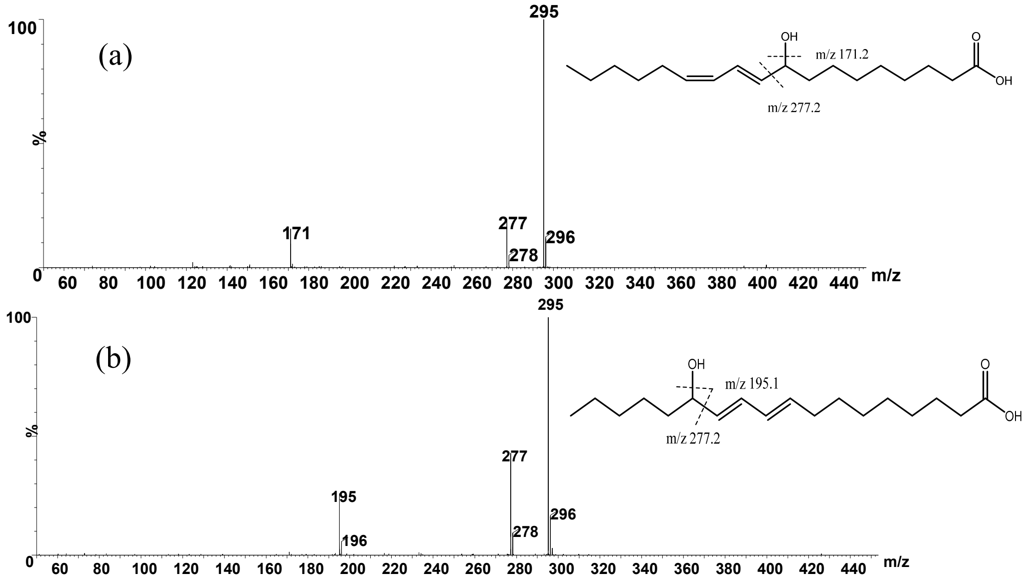

Figure S4. LC/QTOF-MS (ESI⁻) fragmentation pattern of 10(*E*),12(*Z*)-9-hydroxyoctadecadienoic acid (**a**) and 9(*E*),11(*E*)-13-hydroxyoctadecadienoic acid (**b**).

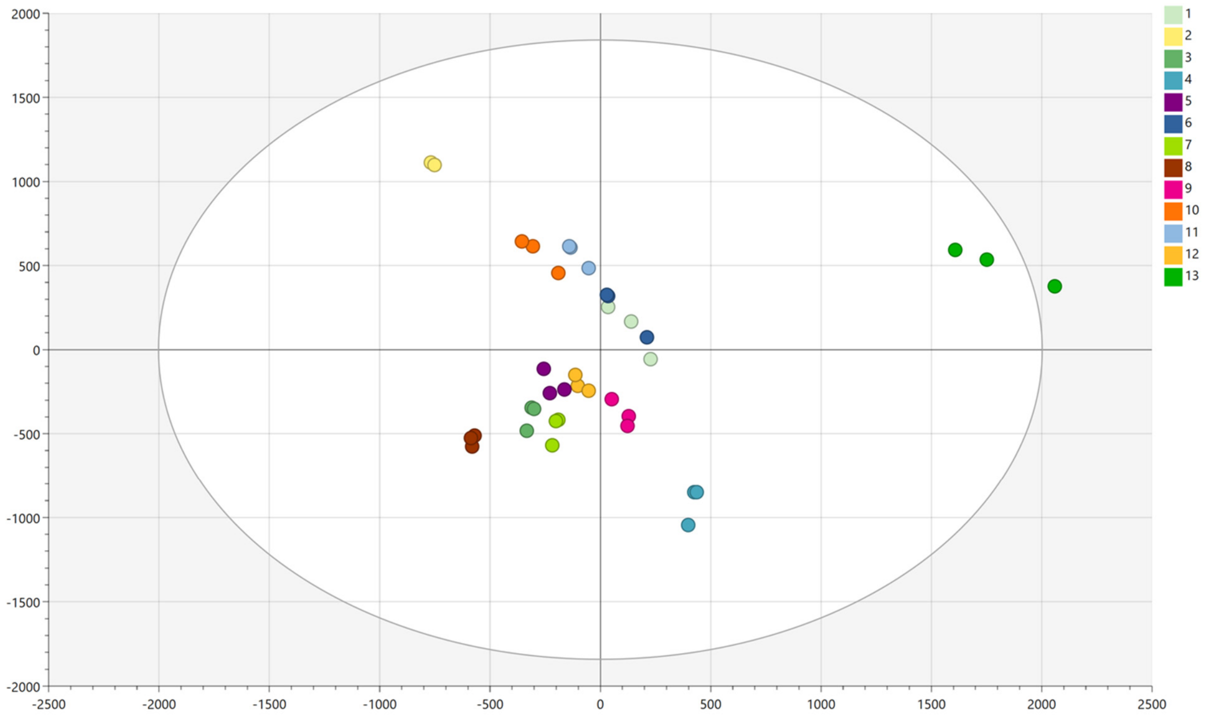

Figure S5. Scores scatter plot of PCA model (Pareto scaling) for LC/MS chemical profiling data (*n* = 791) from thirteen whole wheat bread samples in triplicate. Colored by sample.
